# Metabolic Syndrome During Perinatal Period in Sows and the Link With Gut Microbiota and Metabolites

**DOI:** 10.3389/fmicb.2018.01989

**Published:** 2018-08-24

**Authors:** Chuanshang Cheng, Hongkui Wei, Huichao Yu, Chuanhui Xu, Siwen Jiang, Jian Peng

**Affiliations:** ^1^Department of Animal Nutrition and Feed Science, College of Animal Science and Technology, Huazhong Agricultural University, Wuhan, China; ^2^Key Lab of Agricultural Animal Genetics, Breeding, and Reproduction of Ministry of Education and Key Lab of Swine Genetics and Breeding of Ministry of Agriculture, Huazhong Agricultural University, Wuhan, China; ^3^The Cooperative Innovation Center for Sustainable Pig Production, Wuhan, China

**Keywords:** sow, illumina sequencing, gut microbiota, metabolic syndrome, pregnancy and lactation, *Fusobacterium*

## Abstract

In humans, the metabolic and immune changes occurring during perinatal period also describe metabolic syndrome. Gut microbiota can cause symptoms of metabolic syndrome in pregnant women. Increased gut permeability is also involved in metabolic disorders in non-pregnant hosts. However, longitudinal studies investigating the changes in metabolic characteristics, gut microbiota, and gut permeability of sows throughout pregnancy and lactation are lacking. The correlation between gut microbiota and metabolic status of sows is also poorly known. The present study was conducted to investigate the temporal variations in sow metabolic characteristics, gut microbiota, gut permeability, and gut inflammation at days 30 (G30) and 109 (G109) of gestation and days 3 (L3) and 14 (L14) of lactation. Results showed that insulin sensitivity was decreased in L3. Circulating concentrations of pro-inflammatory cytokine IL-6 increased in G109 and L3. 16S rRNA gene sequencing of the V3-V4 region showed that gut microbiota changed dramatically across different reproductive stages. The bacterial abundance and alpha diversity in L3 were the lowest. The phyla *Proteobacteria* and *Fusobacteria* exhibited the highest relative abundance in L3. Among the genera, *Bacteroides*, *Escherichia_Shigella*, and *Fusobacterium* were highest, but *Oscillospira* the lowest, in relative abundance in L3. The fecal levels of acetate and total short-chain fatty acids were increased in G109, but fecal butyrate concentrations were markedly decreased in L3. The plasma zonulin concentrations, a biomarker for gut permeability, were increased in G109 and L3. The plasma endotoxin concentrations were increased in L3. Furthermore, levels of fecal lipocalin-2 and pro-inflammatory cytokines IL-6 and TNF-α were increased in G109 and L3. In contrast, fecal levels of anti-inflammatory cytokine IL-10 were significantly decreased in G109 and L3. Additionally, the increased relative abundances of *Fusobacterium* in L3 were positively correlated with plasma zonulin and fecal endotoxin but negatively correlated with fecal IL-10. These findings indicate that the mother sow exhibits a metabolic syndrome and dramatical changes in gut microbiota during perinatal period, especially in early lactation. Besides, increased gut permeability and plasma endotoxin concentrations caused by negative microbial changes would possibly be the potential mechanisms under which sow’s metabolic disorders and inflammatory status were exacerbated during early lactation.

## Introduction

The immunological and metabolic statuses of breeding sows directly affect the overall productivity of porcine operations. During normal, healthy pregnancy and lactation, the female body undergoes substantial immunological and metabolic changes (Luan et al., 2014; Nair et al., 2017). These changes are also described as metabolic syndrome including reduced insulin sensitivity in late pregnancy (Barbour et al., 2007). Reduced insulin sensitivity has been correlated with changes in the immune status in pregnancy, including elevated levels of circulating pro-inflammatory cytokines that are thought to drive obesity-associated metabolic inflammation (Saltiel and Olefsky, 2017). In contrast to the obese state where they are detrimental to health, reduced insulin sensitivity is beneficial for fetal growth and the energetic demands of lactation (Nelson et al., 2010; Abduljalil et al., 2012). However, in sows, the excessive decrease in insulin sensitivity during late pregnancy and lactation may unfortunately result in the decreased lactation feed intake of sows (Père and Etienne, 2007). Therefore, longitudinal studies are needed to investigate the changes in metabolic characteristics of sows throughout pregnancy and lactation.

The gut microbiota plays a critical role in nutrient metabolism, immune development, protection from pathogens, and incidence of many chronic diseases in the host (Sommer and Bäckhed, 2013; Gresse et al., 2017). Recently, Koren et al. (2012) observed dramatic changes in the gut microbiota composition of women; these alterations included the loss of diversity and increased *Proteobacteria* and *Actinobacteria* abundances from the first to the third trimester of pregnancy that also persisted 1 month postpartum. Notably, these pregnancy-induced changes in gut microbiota are similar to those detected in inflammatory bowel disease and obese individuals. Interestingly, when germ-free mice are colonized with third trimester maternal microbiota, the mice demonstrate heightened inflammatory responses and insulin insensitivity. This observation suggests that the gut microbiota alterations may directly influence maternal pregnancy-associated metabolic changes (Koren et al., 2012). However, in breeding sows, information regarding how the gut microbiota varies across the reproductive cycle is limited. Likewise, scant information is known on whether and how the gut microbiota contributes to metabolic changes over the course of normal pregnancy and lactation.

In non-pregnant individuals, increased gut permeability is involved in several disorders associated with low-grade inflammation and insulin resistance, including obesity and type 2 diabetes (Teixeira et al., 2012; Cox et al., 2017). The proposed mechanism is presumed to involve the increased passage of gut microbiota components, such as bacterial lipopolysaccharide (LPS), through the intestinal barrier into the circulation. Subsequently, the increased circulating concentrations of bacterial LPS can lead to metabolic endotoxemia, a potential mediator of low-grade inflammation and subsequent metabolic disorders (Cani et al., 2008; Moreira et al., 2012). Emerging data indicate that dysbiosis in gut microbiota may induce an increase in gut permeability (Cani et al., 2009; Shen et al., 2013). Moreover, initial evidence suggests that gut permeability and circulating concentrations of endotoxin were increased in healthy pregnant women relative to those of non-pregnant women (Kerr et al., 2014). However, little is known about the effects of pregnancy and lactation on gut permeability of sows and whether this can lead to subsequent health consequences.

In this study, attempts were made to explore whether breeding sows undergo metabolic syndrome during perinatal period. The work also aimed to evaluate the temporal changes in gut microbiota and their metabolites, gut permeability, and gut inflammation of sows over the course of pregnancy and lactation. The hypothesis of this study is that the mother sow exhibits metabolic syndrome and dramatic changes in gut microbiota during perinatal period and that increased gut permeability and plasma endotoxin concentrations caused by negative microbial changes lead to sow’s metabolic disorders.

## Materials and Methods

### Animals, Diets, and Sample Collection

All procedures involving animals were carried out in accordance with guidelines for animal studies issued by the Institutional Animal Care and Use Committee of Huazhong Agricultural University and its protocol approved by this committee (permit number: HZAUSW-2016-009). A total of twelve multiparous Landrace sows with an average parity of 4.95 ± 1.00 were selected. The sows had no diarrhea or other digestive disorders and did not undergo medication before the study. The sows were allowed to consume the same gestation and lactation diets *ad libitum*. Details are provided in the **Supplementary Material** describing sow diet ingredients and nutrient composition (**Supplementary Table 1**). All diets did not contain any probiotics, antibiotics, or other medicine. Pregnant sows were housed individually in gestation stalls. The sows were moved from the gestation stalls to the farrowing rooms on day 107 of gestation. The sows and piglets were then individually housed in farrowing pens with crates, slatted floors, and heat pads for the piglets. The sows and piglets had free access to water.

Fresh feces were collected directly by massaging the rectum of each sow on days 30 (G30) and 109 (G109) of gestation and days 3 (L3) and 14 (L14) of lactation. Then, 48 samples stored in dry ice were transported to the laboratory and then stored at -80°C until analysis. Three meal tests were conducted at three physiological stages, namely, days 30 and 109 of gestation and day 3 of lactation. Each test was started in the morning after an overnight fasting period of 16–18 h. Blood samples (5 mL) obtained from ear veins were collected 5 min before each meal and 15, 30, 60, 90, 120, 180, and 240 min after the start of each meal for the analysis of glucose values. Blood samples for zonulin, endotoxin, and inflammatory cytokine analyses were collected in heparinized tubes (5 mL) from sows before feeding on days 30 and 109 of gestation and on days 3 and 14 of lactation. Plasma samples were obtained by centrifuging the blood samples at 3000 ×*g* for 10 min at 4°C and then stored at -80 °C until analysis.

### Analysis of Fecal Short-Chain Fatty Acids (SCFAs)

The SCFAs concentrations of feces were analyzed through a gas chromatographic method previously described by Bosch et al. (2008). Briefly, approximately 1.5 g of feces were first homogenized in 1.5 mL of deionized water. The samples were centrifuged at 15,000 ×*g* at 4°C for 10 min. Supernatants (1 mL each) were then acidified with 25% metaphosphoric acid at a 1:5 ratio (1 volume of acid for 5 volumes of sample) for 30 min on ice. The sample was injected into a GC 2010 series gas chromatograph (Shimadzu, Japan) equipped with a CP-Wax 52 CB column 30.0 m × 0.53 mm i.d (Chrompack, Netherlands). The injector and detector temperatures were 75 and 280°C, respectively. Total SCFAs were determined as the sum of analyzed acetate, propionate, and butyrate. Additionally, three-branched fatty acids, namely, isobutyrate, isovalerate, and valerate were measured. All procedures were performed in triplicate.

### Metabolic Biomarker Analyses

Several metabolic biomarkers related to gut health and body insulin sensitivity were measured in the animal molecular nutrition laboratory. These biomarkers included fecal endotoxin, plasma zonulin, and endotoxin as markers for gut permeability (Cani et al., 2008; Tripathi et al., 2009); plasma TNF-α and IL-6 as markers for systemic immune system activation (Bethin et al., 2000); fecal lipocalin-2 and a set of fecal cytokines as markers for gut local inflammation (Chassaing et al., 2012; Koren et al., 2012); and plasma glucose before and after meal as markers for insulin sensitivity (Père et al., 2000). Zonulin, endotoxin, lipocalin-2, IL-6, IL-10, and TNF-α concentrations in feces or plasma were determined by using sow enzyme-linked immunosorbent assay kits (Bio-Swamp, Wuhan, China) according to the manufacturer’s instructions. Plasma glucose was determined with a glucose dehydrogenase activity colorimetric assay kit (BioVision Inc., CA, United States). Values were obtained by measuring the OD_450_ by using a Labsystems Multiskan MS microplate reader (Vantaa, Finland). All procedures were performed in triplicate.

### DNA Extraction, 16S rDNA Amplification, and Illumina Miseq Sequencing

Microbial DNA was extracted from 220 mg of each fecal sample with a QIAamp Fast DNA Stool Mini Kit (Qiagen, Germany) according to the manufacturer’s instructions. Successful DNA isolation was separated by agarose gel electrophoresis. The forward primer 341F (5′-ACTCCTACGGGAGGCAGCAG-3′) and the reverse primer 806R (5′-GGACTACHVGGGTWTCTAAT-3′) were used for the amplification of the V3–V4 hypervariable region of an 16S rRNA gene. The PCR conditions were pre-denaturation cycle at 94°C for 4 min, 25 cycles of denaturation at 94°C for 30 s, annealing at 50°C for 45 s, elongation at 72°C for 30 s, and a final post-elongation cycle at 72°C for 5 min. The PCR products were purified with AmpureXP beads (AGENCOURT). After purification, the PCR products were used for the construction of the libraries and then paired-end sequenced (2 × 250) on a MiSeq platform (Illumina, United States) at the Beijing Genomics Institute (BGI, China).

### Sequence Filtering, OTU Clustering, and Sequence Analyses

Sequences with an average Phred score of lower than 30, ambiguous bases, homopolymer runs exceeding 6 bp, primer mismatches, and sequence lengths shorter than 100 bp were removed. Only sequences with overlaps longer than 10 bp and without any mismatch were assembled according to their overlap sequences. Reads that failed to assemble were discarded. Barcode and sequencing primers were trimmed from the assembled sequence. To avoid the effect of the sequencing depth on the composition of microbiota (Hughes and Hellmann, 2005), we rarefied the library size to 27871 tag-depth per sample by using the rarefy function in R package. High-quality tags were clustered into operational taxonomic units (OTUs) at the 97% similarity with the USEARCH (Edgar, 2010). Taxonomy assignments for 16S rRNA gene sequences were performed with the RDP classifier program (V2.2) (Wang et al., 2007) and the Silva 16S sequence database. A Venn diagram was generated for comparison among the OTUs of the groups. The alpha diversity values of each sample were assessed on the basis of the observed OTUs, abundance-based coverage estimator (ACE), bias-corrected Chao richness estimator (Chao 1), Shannon index, and Simpson index. Beta diversity measures depended on Bray-Curtis distance were calculated using mothur. Linear discriminant analysis coupled with effect size (LEfSe) was conducted to identify bacterial taxa differentially represented between different stages at genus or higher taxonomy levels (Segata et al., 2011).

### Statistical Analyses

Each sow was considered an experimental unit in all statistical analyses. Before analysis, Kolmogorov–Smirnov and Levene tests were performed for the normality and heteroscedasticity of the data (with the significance level set at 5%). Data on SCFAs, lipocalin-2, glucose, zonulin, endotoxin, and cytokines values were assessed by ANOVA. The procedure for the repeated measurements of SAS (SAS Institute, Inc., Cary, NC, United States) was used. Data were given as means ± standard errors of the means. A mean comparison was performed using the Duncan’s Multiple Range test method (Duncan, 1955) with a significant level of *P* < 0.05.

We used a nonparametric Mann–Whitney test to determine the variance of alpha diversity indices and relative abundance of gut microbiota. Structural variations between interindividual and intraindividual were tested by student’s *t*-test with 1000 Monte Carlo permutations based on Bray-Curtis and weighted UniFrac distances. Correlations were analyzed by using Spearman’s correlation in R 3.0.2 with the Rstudio 0.97.310 package and gplots package for the heat map. Differences were considered statistically significant when *P*-value < 0.05. The data were corrected by false discovery rate analysis according to the Benjamini–Hochberg method, with an α of < 0.05 (Benjamini and Hochberg, 2000).

## Results

### During the Perinatal Period, Especially in Early Lactation, Sows Showed Obvious Metabolic Syndrome

**FIGURE 1:**
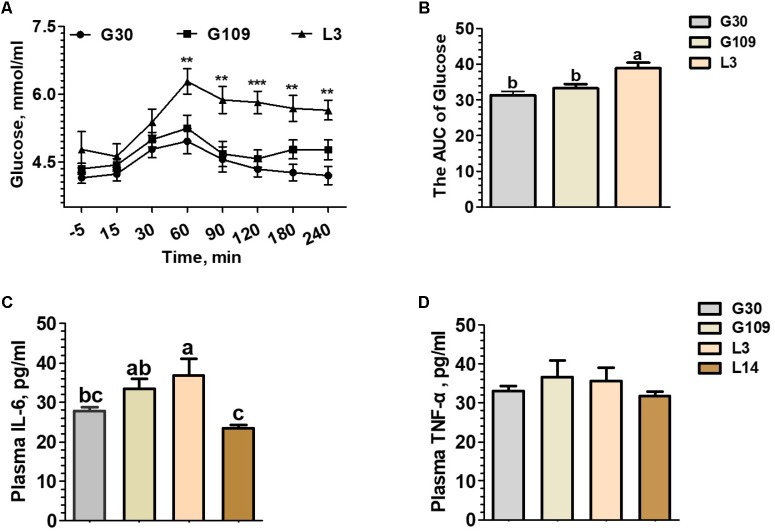
During the perinatal period, especially in early lactation, sows showed obvious metabolic syndrome. Effects of reproductive stages on plasma concentrations of glucose **(A)** and the area under curve (AUC) of plasma glucose after meal tests **(B)** and plasma concentrations of IL-6 **(C)** and TNF-α **(D)**. Data are presented as means ± SEM (*n* = 12). a,b, Significant effect of sampling day (values with different lowercase letters are significantly different). G109, day 109 of gestation; L3, day 3 of lactation. ^∗∗^*P* < 0.01; ^∗∗∗^*P* < 0.0001. a–c, significant effect of sampling day (*P* < 0.05; values with different lowercase letters are significantly different).

To determine whether the metabolic characteristics change over the course of a normal pregnancy and lactation in sows, the concentrations of plasma glucose before and after meal and circulating pro-inflammatory cytokines (TNF-α and IL-6) were assessed. Between 60 and 240 min after meals, L3 showed higher plasma glucose concentrations than those of G30 or G109 (**Figure 1A**). Furthermore, the area under curve of the plasma glucose levels were remarkly higher in L3 than in G30 or G109 (**Figure 1B**). Although no significant difference in plasma TNF-α level was noted (**Figure 1D**), the plasma IL-6 levels in G109 and L3 were markedly higher than those in G30 and L14 (**Figure 1C**). Taken together, the results supported that the sow’s body exhibits a reduce in insulin sensitivity and develops a low-grade inflammation during early lactation.

### The Gut Microbiota Composition of Sows Is Profoundly Altered During Pregnancy and Lactation

**FIGURE 2:**
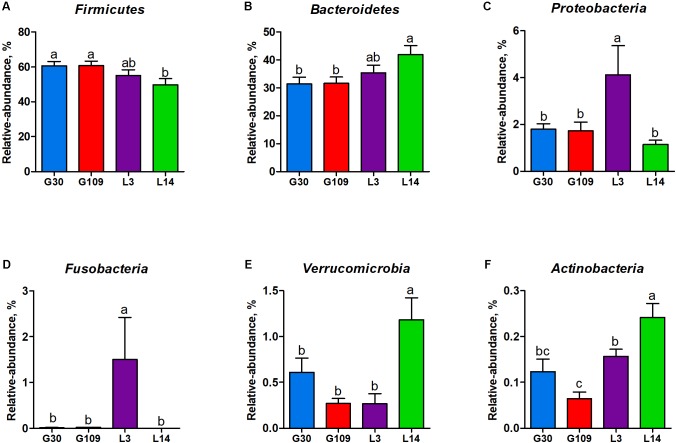
Changes in the five distinct bacterial phyla in sow gut across the reproductive cycle. **(A)** Firmicutes, **(B)** Bacteroidetes, **(C)** Proteobacteria, **(D)** Fusobacteria, **(E)** Verrucomicrobia, and **(F)** Actinobacteria. Data are presented as means ± SEM (*n* = 12). a–c, significant effect of sampling day (*P* < 0.05; values with different lowercase letters are significantly different). G109, day 109 of gestation; L3, day 3 of lactation

**FIGURE 3:**
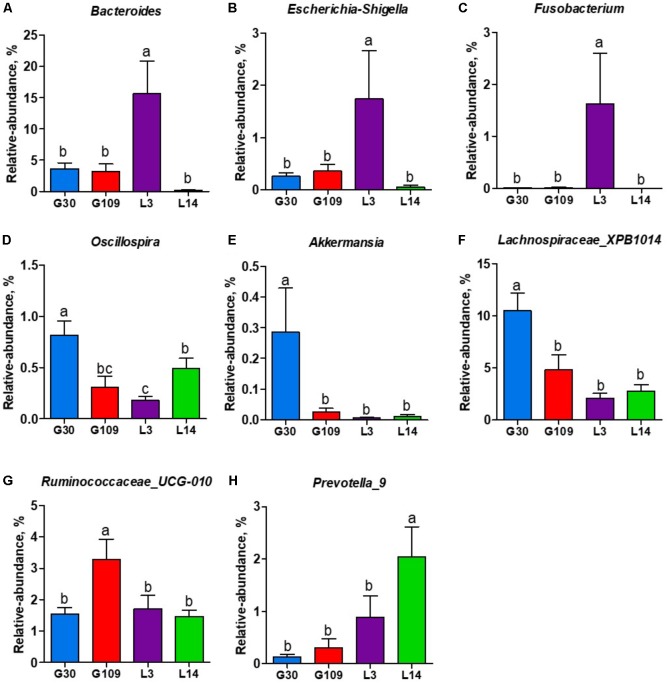
The changes of 7 distinct genera in the gut microbiota composition of sows across the reproductive cycle. **(A)** *Bacteroides*, **(B)** *Escherichia-Shigella*, **(C)** *Fusobacterium*, **(D)** *Oscillospira*, **(E)** *Akkermansia*, **(F)** *Lachnospiraceae_XPB1014*, **(G)** *Ruminococcaceae_UCG-010*, and **(H)** *Prevotella_9*. Data are presented as means ± SEM (*n* = 12). a–c, significant effect of sampling day (values with different lowercase letters are significantly different). G109, day 109 of gestation; L3, day 3 of lactation.

**FIGURE 4:**
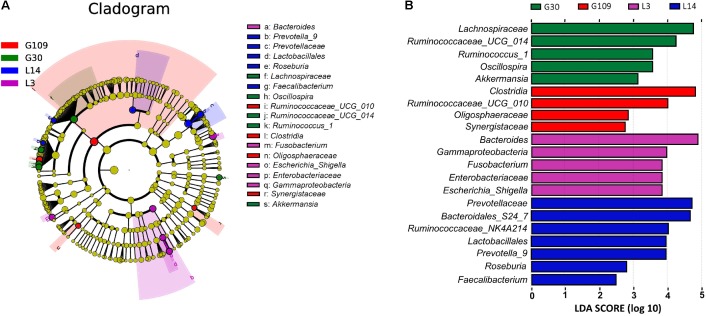
LEfSe analysis of the gut microbiota composition of sows across the reproductive cycle. **(A)** Cladogram using LEfSe method indicating the phylogenetic distribution of gut microbiota in sows across the reproductive cycle. Each successive circle represents a phylogenetic level. **(B)** Histogram of the LDA scores reveals the most differentially abundant taxa among different reproductive stage. G109, day 109 of gestation; L3, day 3 of lactation.

All 48 fecal samples were subjected to 16S rRNA gene sequencing. Illumina Miseq sequencing of the V3–V4 regions of bacterial 16S rRNA genes generated 1 333 411 high-quality sequences. On the basis of 97% sequence similarity, we obtained 6047 OTUs, 3131 of which existed in all groups and were thus defined as core OTUs (**Supplementary Figure 1**). The core OTUs comprised 51.8% of the total OTUs, whereas 78, 146, 86, and 161 OTUs were uniquely identified at G30, G109, L3, and L14, respectively (**Supplementary Figure 1**). The OTUs were classified into 15 bacterial phyla and 231 genera. The top 10 phyla and top 10 genera in relative abundance of fecal microbiota present in sows are displayed in **Supplementary Figure 2**. *Firmicutes* (56.58%) and *Bacteroidetes* (35.07%) were the most dominated phyla in sows, followed by *Spirochaetes* (3.40%) and *Proteobacteria* (2.20%). At the genus level, *Eubacterium_coprostanoligenes* (6.47%), *Bacteroides* (5.68%), *Prevotellaceae_NK3B31* (5.45%), *Lachnospiraceae_XPB1014* (5.04%), *Christensenellaceae_R-7* (4.73%), *Rikenellaceae_RC9_gut* (3.92%), *Treponema_2* (3.07%), *Clostridium_sensu_stricto_1* (2.87%), *Lachnospiraceae_NK4A136* (2.82%), and *Ruminococcaceae_UCG-005* (2.61%) were the 10 most abundant genera. Significant differences in the levels of phylum and genus of fecal microbiota among the four breeding stages were further identified. The relative abundance of the phylum Firmicutes was higher in G30 and G109 than in L14 (**Figure 2A**). However, the relative abundance of *Bacteroidetes* was lower (*P* < 0.05) in G30 and G109 than in L14 (**Figure 2B**). In addition, the relative abundance of *Proteobacteria* and *Fusobacteria* was the highest (*P* < 0.05) in L3 and *Verrucomicrobia* and *Actinobacteria* were the highest (*P* < 0.05) in L14 (**Figures 2C–F**). At the genus level, the relative abundances of *Bacteroides*, *Escherichia Shigella* and *Fusobacterium* were the highest but that of *Oscillospira* was the lowest in L3 (**Figures 3A–D**). Akkermansia, which was abundant in the gut of the G30 sows, was nearly absent in the G109, L3, and L14 sows (**Figure 3E**). Furthermore, the mean relative abundances of Lachnospiraceae_XPB1014, Ruminococcaceae_UCG-010, and Prevotella_9 were the highest in G30, G109, and L14, respectively (**Figures 3F–H**). Of the above genera, the bacterial abundances of 26 genera changed across different reproductive stages (**Supplementary Table 2**). The microbial compositions among the four breeding stages were further analyzed using the LefSe (**Figure 4**). Our LEfSe analysis revealed that Lachnospiraceae, Ruminococcus_1, Oscillospira, and Akkermansia were enriched in G30 sows; Clostridia and Ruminococcaceae_UCG_010 were enriched in G109 sows; Gammaproteobacteria, Fusobacterium, Enterobacteriaceae, and Escherichia_Shigella were enriched in L3 sows; Ruminococcaceae_NK4A214, Lactobacillales, Roseburia, and Faecalibacterium were enriched in L14 sows (**Figures 4A,B**). Therefore, the above results indicate that the gut microbiota composition of sows is profoundly altered during pregnancy and lactation.

### The Gut Microbiota Diversity of Sows Is Significantly Altered During Pregnancy and Lactation

**FIGURE 5:**
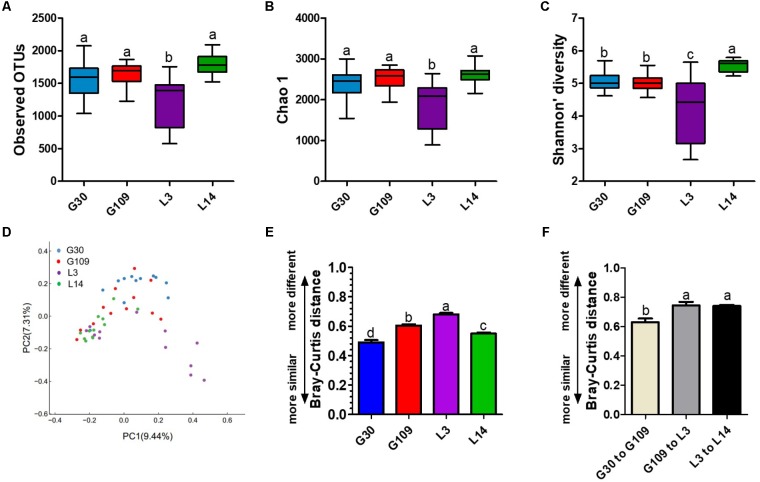
The gut microbiota diversity of sows is significantly altered during pregnancy and lactation. **(A)** Comparisons of the number of observed OTUs among different reproductive stage sows. **(B)** Comparisons of bias-corrected Chao richness estimator (Chao 1) among different reproductive stage sows. **(C)** Comparisons of Shannon diversity indices among different reproductive stage sows. **(D)** Trajectory of the gut microbiota structure of sows across reproductive cycle based on Bray–Curtis distance. **(E)** Interindividual variations were determined by average Bray–Curtis distances between individuals in day 30 (G30) and 109 (G109) of gestation and day 3 (L3) and 14 (L14) of lactation, respectively. **(F)** Intraindividual variations were determined by distances between paired G30 and G109, G109 and L3, and L3 and L14 samples, respectively. Data are presented as means ± SEM (*n* = 12). a–d, significant effect of sampling day (*P* < 0.05; values with different lowercase letters are significantly different). G109, day 109 of gestation; L3, day 3 of lactation.

Given the remarkable changes in composition of the gut microbiota of the sows across different breeding stages, we assessed the alpha and beta diversity of the fecal microbiota. The observed OTUs, Chao 1, ACE, and Shannon index were lowest in L3 (**Figures 5A–C** and **Supplementary Figure 3A**). No remarkable difference was noted in the observed OTUs, Chao 1, and ACE among the G30, G109, and L14 sows. The Shannon index in L14 was higher than that in G30 and G109. In addition, the Simpson index in L3 was higher than that in G30, G109, or L3 (**Supplementary Figure 3B**). The dramatic changes in the alpha diversity of gut microbiota in sows across different breeding stages enabled us to assess the extent of interindividual and intraindividual structural variations of gut microbiota in sows. Through principal coordinate analysis (PCoA) based on Bray–Curtis distance, we found that the gut microbiota of sows were less dispersed in G30 and L14 but showed obvious segregation in G109 and L3 (**Figure 5D**). Moreover, the results of the PCoA based on weighted UniFrac distance revealed a similar shifting pattern (**Supplementary Figure 4A**). The interindividual Bray–Curtis distances among different individuals increased significantly from G30 to L3 and then decreased markedly from L3 to L14 (**Figure 5E**). Moreover, the dramatic intraindividual variations from G109 to L3 and from L3 to L14 were significantly higher than that from G30 to G109 (**Figure 5F**). Such structural variations were also confirmed by weighted UniFrac distance (**Supplementary Figure 4B**). Taken together, the gut microbiota diversity of sows is significantly altered during pregnancy and lactation.

### The Gut Microbiota Metabolites of Sows Were Significantly Altered During Pregnancy and Lactation

**FIGURE 6:**
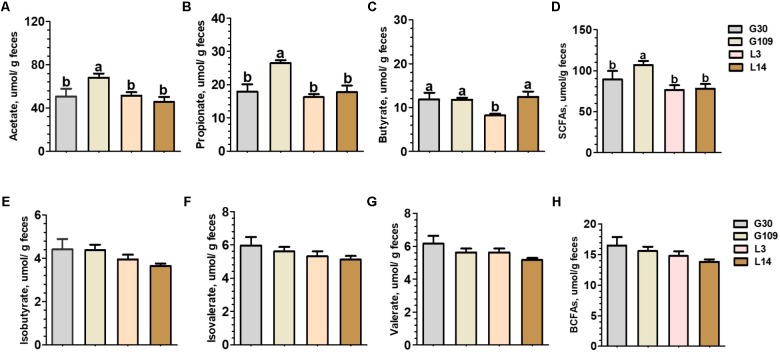
Fecal concentrations of intestinal microbial metabolites in sows during gestation and lactation. **(A)** acetate, **(B)** propionate, **(C)** butyrate, **(D)** SCFAs, **(E)** isobutyrate, **(F)** isovalerate, **(G)** valerate, and **(H)** BCFAs. Data are presented as means ± SEM (*n* = 12). a,b, significant effect of sampling day (values with different lowercase letters are significantly different). G109, day 109 of gestation; L3, day 3 of lactation. SCFAs, short-chain fatty acid; BCFAs, branch-chain fatty acids. SCFAs is the sum of acetate, propionate, and butyrate; BCFAs is the sum of isobutyrate, isovalerate, and valerate.

Significant changes in gut microbial composition may cause changes in their metabolites. Therefore, we observed the changes of several SCFAs in feces of sows at different breeding stages. The fecal level of acetate, propionate, and total SCFAs were significantly higher in G109 than those in G30, L3, or L14 (**Figures 6A,B,D**). Interestingly, the lowest fecal concentrations of butyrate were observed in L3 (**Figure 6C**). No significant differences with respect to the fecal levels of isobutyrate, isovalerate, and valerate and total BCFAs were found among the four breeding stages (**Figures 6E–H**).

### Gut Permeability and Plasma Endotoxin Concentrations of Sows Were Increased in Early Lactation

**FIGURE 7:**
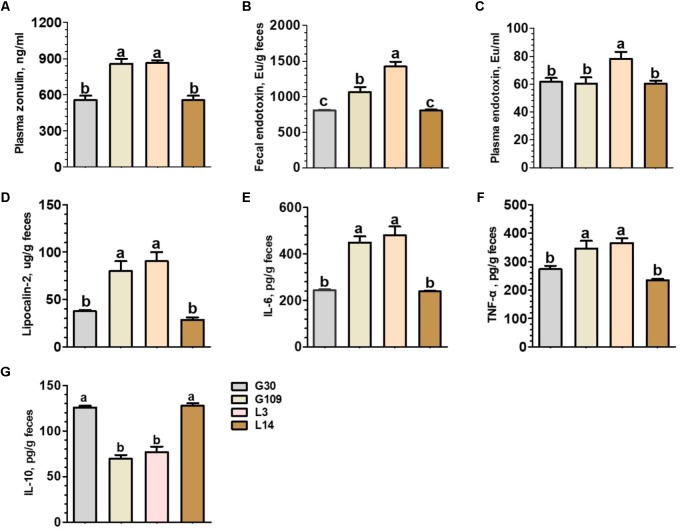
Plasma concentrations of zonulin **(A)** and endotoxin **(C)** and fecal concentrations of endotoxin **(B)**, Lipocalin-2 **(D)**, IL-6 **(E)**, TNF-α **(F)**, and IL-10 **(G)** of the sows during gestation and lactation. Data are presented as means ± SEM (*n* = 12). a–c, significant effect of sampling day (values with different lowercase letters are significantly different). G109, day 109 of gestation; L3, day 3 of lactation.

Considering recent evidence suggesting that microbiota-derived butyrate modulate intestinal barrier integrity via modulation of expression of tight junction proteins (Kelly et al., 2015), we investigated whether gut permeability changes in the course of a normal pregnancy and lactation in sows. The results presented in **Figure 7A** showed that the plasma levels of zonulin were significantly increased in G109 and L3 than in G30 and L14. Previous studies have suggested that the impaired intestinal mucosal integrity may enhance concentrations of zonulin in blood (Fasano, 2011). This finding suggests that gut permeability of sows were increased during the perinatal period. We next detected the endotoxin in feces and plasma of sows at different breeding stages. The results showed that the concentrations of fecal endotoxin increased dramatically from G109 to L3 and then decreased in L14 (**Figure 7B**). The highest plasma endotoxin concentration (*P* < 0.05) was found in L3 (**Figure 7C**).

### A Low-Grade Inflammation Developed During Perinatal Period at the Intestinal Mucosal Epithelium of Sows

In non-pregnant individuals, increased gut permeability has been associated with an increased risk of systemic or local inflammation (Teixeira et al., 2012). However, little is know about the effects of pregnancy and lactation on the local inflammatory response within the intestine of sows. To address this question, we measured four biomarkers for inflammation in the intestine. These biomarkers included fecal lipocalin-2 (Chassaing et al., 2012) and a set of fecal cytokines (IL-6, IL-10, and TNF-α) (Saiki et al., 1998). The levels of fecal lipocalin-2 and the proinflammatory cytokines IL-6 and TNF-α were significantly higher in G109 and L3 than in G30 and L14 (**Figures 7D–F**). In contrast, the levels of anti-inflammatory cytokine IL-10 were significantly lower in G109 and L3 than in G30 and L14 (**Figure 7G**). Therefore, our data suggest that the mucosal surfaces of the intestine in sows present low-grade inflammation during perinatal period.

### Correlations Between Gut Microbiota and Parameters of Gut Permeability and Gut Inflammation in Sows

**FIGURE 8:**
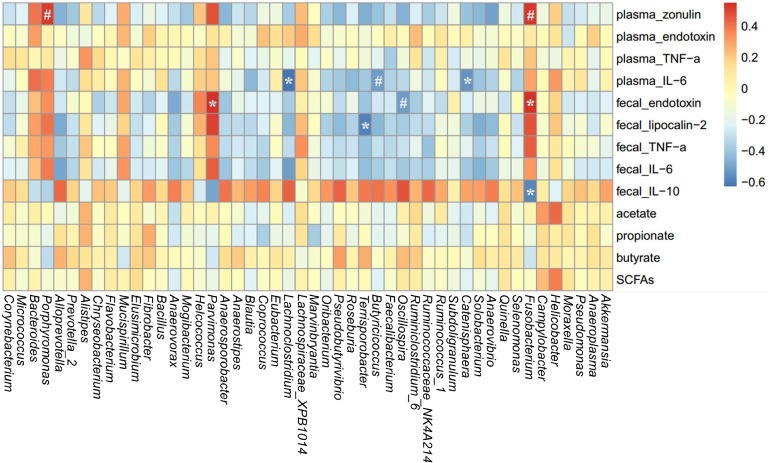
Heatmap of the spearman r correlations between the gut microbiota significantly modified by different reproductive stages and metabolic parameters of sows. Data are presented as means ± SEM (*n* = 12). ^∗^*P* < 0.05; ^#^*P* < 0.1 (following the Spearman correlation analysis). SCFAs is the sum of acetate, propionate, and butyrate.

A spearman correlation analysis was performed to evaluate the potential link between alterations in gut microbiota composition and the parameters of gut permeability and gut inflammation in sows (**Figure 8**). The genus *Fusobacterium* was positively correlated with plasma zonulin (*r* = 0.53, *P* < 0.05) and fecal endotoxin (*r* = 0.54, *P* < 0.05) but negatively correlated with fecal IL-10 (*r* = –0.57, *P* < 0.05). *Lachnoclostridium* (*r* = –0.62, *P* < 0.05), *Catenisphaera* (*r* = –0.54, *P* < 0.05), and *Butyricicoccus* (*r* = –0.52, *P* = 0.088) was negatively correlated with plasma IL-6. Additionally, *Parvimonas* was positively correlated with fecal endotoxin (*r* = 0.55, *P* < 0.05), and *Terrisporobacter* was negatively correlated with fecal lipocalin-2 (*r* = -0.59, *P* < 0.05).

## Discussion

The immunological and metabolic status of breeding sows directly affect the overall productivity of procine operations. However, longitudinal studies investigating changes in the metabolic characteristics of sows throughout pregnancy and lactation are lacking. In the current study, the breeding sows were found to suffer from symptoms of metabolic syndrome including low-grade inflammation during perinatal period and reduced insulin sensitivity in early lactation. This pro-inflammatory environment in late pregnancy may promote the contraction of the uterus, expulsion of the piglets and rejection of the placenta (Mor and Cardenas, 2010). Furthermore, elevated levels of circulating pro-inflammatory cytokines have been reported for late pregnancy and have been correlated with levels of insulin resistance (Mor and Cardenas, 2010). The increased insulin resistance in mother sows also results in elevated insulin-mediated glucose and free fatty acid levels, allowing for greater substrate availability for newborn piglets growth (Girard et al., 1992). However, in sows, an excessive decrease in insulin sensitivity during early lactation may unfortunately result in decreased lactation feed intake of sows (Père and Etienne, 2007). A decrease in feed intake during lactation directly causes sow to reduce milk volume and increase body loss, which will affect the growth performance of piglets and prolong the interval from weaning to estrus (Koketsu et al., 1996; Eissen et al., 2003). Thus, our findings suggest that pig producers need to attach importance to monitoring and regulation of insulin sensitivity during early lactation.

The cause of reduced insulin sensitivity in lactation remains unclear. In the case of non-pregnant obesity, recent studies have suggested a role for gut microbiota in driving metabolic diseases, including inflammation and reduced insulin sensitivity (Boulangé et al., 2016; Saad et al., 2016). In addition, Koren et al. (2012) observed dramatic changes in gut microbiota composition of women from the first to the third trimester of pregnancy, whereas the gut microbiota alterations directly influence maternal pregnancy-associated metabolic changes. These studies prompted us to investigate the effects of pregnancy and lactation on sow’s gut microbiota and its link with metabolic syndrome in sows. The results suggest the emergence of a dramatic change in gut microbiota of sows over the course of pregnancy and lactation. The L3 sows exhibited the lowest gut microbial richness and alpha diversity. Moreover, the between-individual diversity increased from G30 to L3 and then decreased from L3 to L14. This finding is consistent with the result of Koren et al. (2012), who found that gut microbiota diversity and richness decreased in 1 month of postpartum in women. Recent studies have shown a correlation between low gut microbiota richness and adverse metabolic conditions, including insulin resistance and a more pronounced inflammatory phenotype (Le Chatelier et al., 2013). Moreover, Mokkala et al. (2016) suggests that low gut microbiota richness is associated with increased gut permeability in overweight pregnant women. Similarly, we also observed an increase in gut permeability in early lactation of normal sows. Thus, the interplay between the gut microbiota and gut permeability may be one of the potential mechanism linking the gut microbiota to sow’s metabolic disorders during early lactation.

Besides gut microbiota diversity, the phylogenetic composition of gut microbiota in sows also shifts substantially over the course of pregnancy and lactation. *Firmicutes* and *Bacteroidetes* were the most dominant phyla regardless of breeding stages, as previously suggested (Kong et al., 2016; Chen et al., 2017). In our study, the relative abundance of phylum *Firmicutes* was higher in pregnancy than in lactation, whereas *Bacteroidetes* showed the opposite result. Earlier studies showed that obesity is associated with an increase in *Firmicutes* abundance (Tsai and Coyle, 2009). The increased *Firmicutes* was considered a means of enhancing the body’s capacity for energy acquisition from the diet, a potential advantage to support growth of the fetus and prepare the body for the energy needs of lactation (Turnbaugh et al., 2006). In agreement with this, we observed that the fecal levels of acetate, propionate, and total SCFAs were increased in late pregnancy of sows. Acetate is utilized for lipogenesis in the liver and as a fuel source once it enters the peripheral circulation (Topping and Clifton, 2001). Propionate is largely taken up by the liver, and is used as a substrate for hepatic gluconeogenesis. The increased SCFAs production promotes intestinal energy availability that may contribute to the energetic demands of lactation.

Remarkably, an enrichment of *Proteobacteria* and *Fusobacteria* was observed in L3. Likewise, the genera *Escherichia shigella* (phylum Proteobacteria) and *Fusobacterium* (phylum Fusobacteria) were also enriched in L3. *Proteobacteria* are a minor constituent within a balanced gut-associated microbial community (Eckburg et al., 2005). However, a dysbiotic expansion of facultative anaerobic *Proteobacteria* was observed during conditions of gut inflammation, including irritable bowel syndrome and inflammatory bowel disease (Morgan et al., 2012). Similarly, an increased *Proteobacteria* was observed during late pregnancy in women which induces heightened inflammatory responses and insulin insensitivity in germ-free mice (Koren et al., 2012). Recent studies have proposed that an expansion of *Proteobacteria* is a potential diagnostic microbial signature of dysbiosis in gut microbiota and epithelial dysfunction (Shin et al., 2015; Litvak et al., 2017). In addition to *Proteobacteria*, substantial evidence suggests that *Fusobacterium* is closely correlated with gut inflammation and cancer in humans and other diseases in animals (De Witte et al., 2017; Yang Y. et al., 2017). Neonatal piglet diarrhea is also associated with increases in the relative abundance of *Fusobacterium* (Yang Q. et al., 2017). Therefore, given these observations, we hypothesized that the L3 mucosal surfaces of the intestine in sows may present low-grade inflammation. Consistent with the hypothesis, we observed that levels of fecal lipocalin-2 and the pro-inflammatory cytokines IL-6 and TNF-α were increased in L3. Meanwhile, the genus *Fusobacterium* was shown to be negatively correlated with the anti-inflammatory cytokine IL-10 in feces. Taken together, we suggest that the enrichment of *Proteobacteria* and *Fusobacteria* during early lactation leads to low-grade inflammation at intestinal mucosal epithelium, and this inflammation may drive the microbial dysbiosis into a positive feedback loop with the altered host response. However, the underlying mechanism by which gut microbiota in lactating sows leads to gut inflammation needs further study.

The abundances of health-related bacteria were also impacted in L3. For instance, *Oscillospira*, which is strongly correlated with the formation of secondary bile acids and decreased in abundance in inflammatory diseases (Walters et al., 2014; Konikoff and Gophna, 2016), was less abundant on average in L3. Notably, the relative abundances of butyrate-producing bacteria such as *Ruminococcus*_1 (phylum Firmicutes, family Ruminococcaceae) and *Lachnospiraceae_XPB1014* (phylum Firmicutes, family Lachnospiraceae) were reduced in L3. The decrease in abundance of these two genera coincided with the decreased concentrations of fecal butyrate in L3 sows. Furthermore, we also observed that gut permeability and plasma endotoxin concentrations of sows were increased in early lactation, and a spearman correlation analysis showed that *Fusobacterium* was positively correlated with the biomarker of gut permeability. Recent evidence suggests that microbiota-derived butyrate is essential for the maintenance of intestinal epithelium integrity (Singh et al., 2014; Kelly et al., 2015; Huang et al., 2015). Meanwhile, in non-pregnant individuals, increased gut permeability and circulating concentrations of endotoxin have been associated with several disorders associated with low-grade inflammation and insulin resistance, including obesity and type 2 diabetes (Teixeira et al., 2012; Cox et al., 2017). Thus, based on these observations above, we suggest that the decreased butyrate-producing bacteria *Ruminococcus_1* and *Lachnospiraceae_XPB1014* in early lactation leads to a decrease in butyrate production, which in turn contributes to an increase in gut permeability and endotoxemia. Ultimately, increased endotoxemia triggers low-grade inflammation and subsequent metabolic disorders in sows. However, although these changes occur simultaneously in early lactating sows, the direct link between them remains to be further confirmed.

In conclusion, the present study suggests that the breeding sow undergoes metabolic syndrome including insulin insensitivity and low-grade inflammation during perinatal period, especially in early lactation. The gut microbiota and their metabolites of sows change dramatically over the course of pregnancy and lactation. The representative changes include the expansion of diversity between sows, an increase in *Proteobacteria* and *Fusobacteria*, a decrease in butyrate-producing genera *Ruminococcus_1* and *Lachnospiraceae_XPB1014*, and reduced bacterial richness and butyrate production during early lactation. Besides, increased gut permeability and plasma endotoxin concentrations caused by negative microbial changes would possibly be the potential mechanisms under which sow’s metabolic disorders and inflammatory status were exacerbated during early lactation. Further studies are needed to elucidate the mechanisms underlying the interplay among the gut microbiota, gut permeability, and sow’s metabolic characteristics.

## Accession Number

The 16S rRNA gene sequence information in this study was deposited in NCBI Sequence Read Archive (SRA) under accession number SRP150893.

## Author Contributions

JP conceived and designed the experiments and revised the manuscript. SJ conceived and designed the experiments, analyzed the data, and revised the manuscript. CC performed the experiments, analyzed the data, and wrote the manuscript. HW participated in the research – microbiology and analyzed the data. CX and HY performed the measurement of pig metabolic phenotypes. All authors approved the final version of the manuscript to be published.

## Conflict of Interest Statement

The authors declare that the research was conducted in the absence of any commercial or financial relationships that could be construed as a potential conflict of interest.
