# Supplementary material for: Metabolic Syndrome During Perinatal Period in Sows and the Link With Gut Microbiota and Metabolites
Source: Front Microbiol. 2018 Aug 24;9:1989. doi: 10.3389/fmicb.2018.01989 (PMC6117386; doi:10.3389/fmicb.2018.01989)
Supplement: Supplementary file 1 [file Data_Sheet_1.DOC]

Supplementary Material

Dynamic Distribution of the Gut Microbiota and the Relationship with Metabolic Characteristics during Pregnancy and Lactation in Sows

Chuanshang Cheng, Hongkui Wei, Huichao Yu, Chuanhui Xu, Siwen Jiang* and Jian Peng*

*** Correspondence:** Siwen Jiang: jiangsiwen@mail.hzau.edu.cn;
Jian Peng: pengjian@mail.hzau.edu.cn

# Supplementary Figures and Tables

## Supplementary Figures


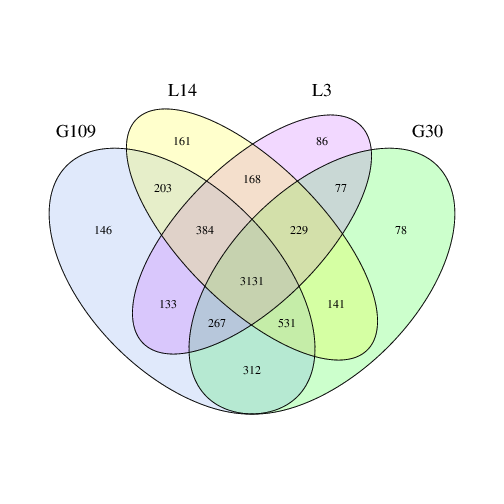


**Supplementary Figure 1.** **A Venn diagram was generated to compare OTUs between the different reproductive stages and to depict OTUs that were unique to the four stages.**

**
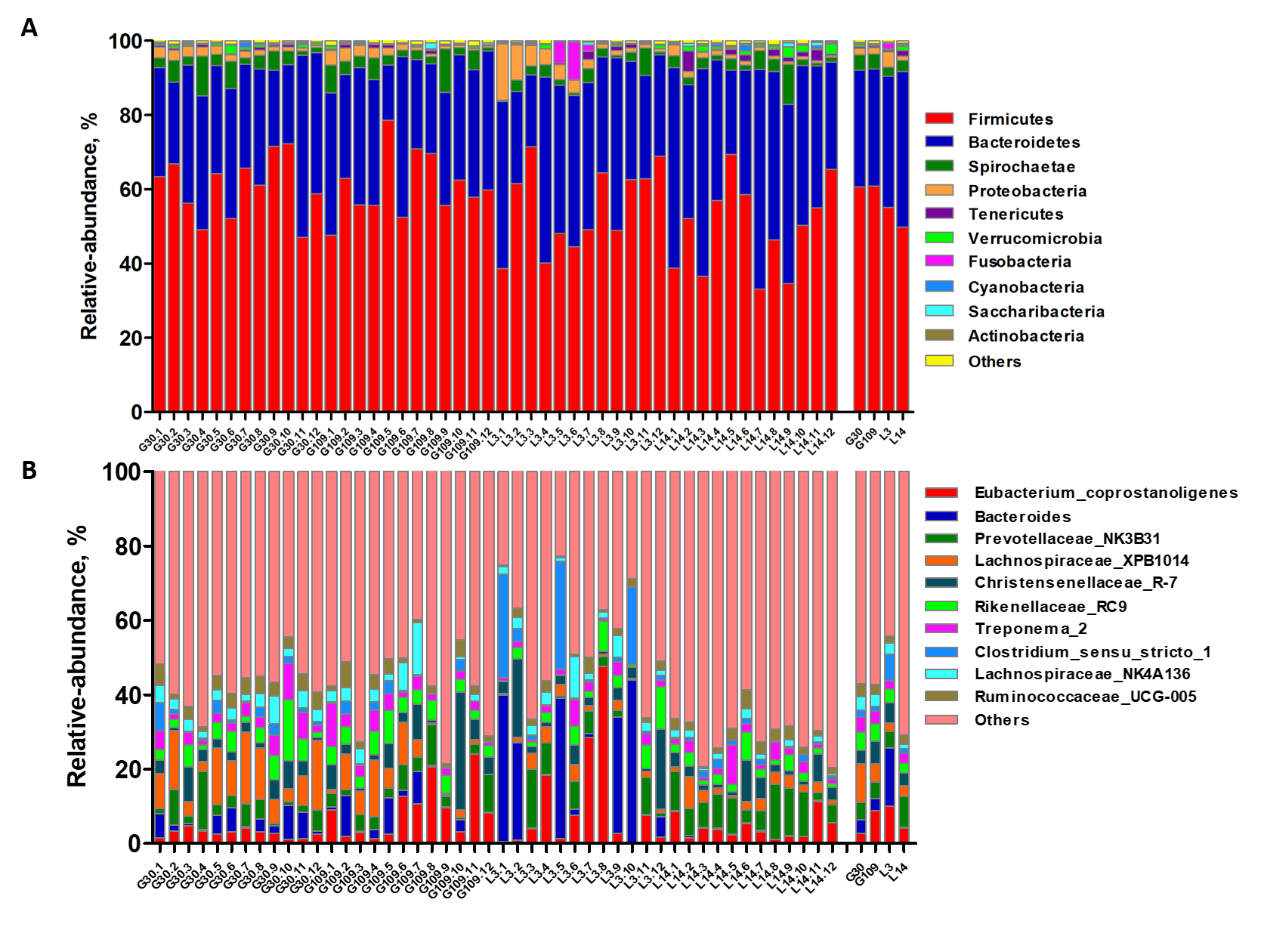
**

**Supplementary Figure 2. Taxonomic distribution of bacterial phyla (A) and genera (B) obtained from 16S rRNA gene sequencing.**


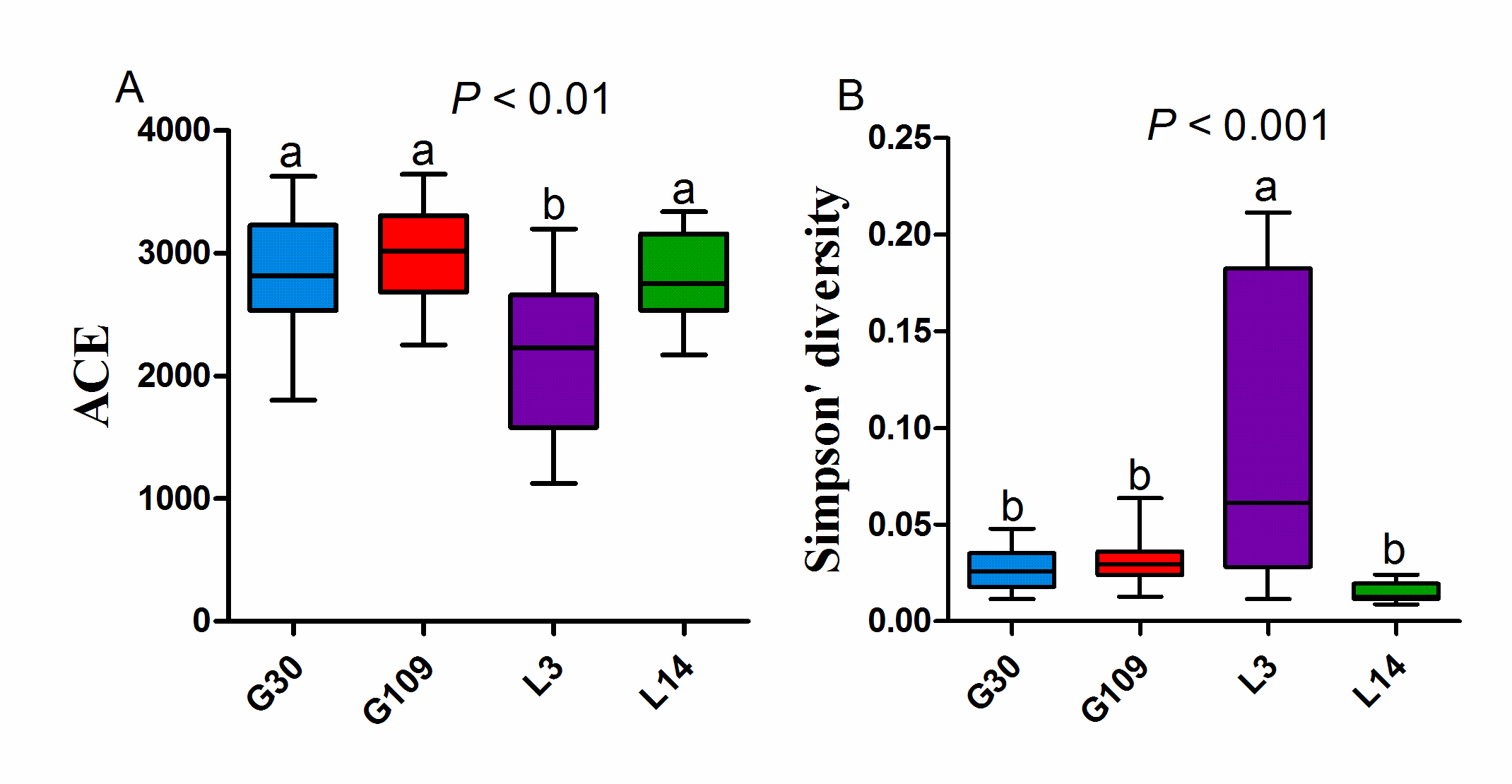


**Supplementary Figure 3. Variations in alpha diversity of the sows during different reproductive stages. (A)** Comparisons of abundance-based coverage estimator (ACE) between different reproductive stages in sows. **(B)** Comparisons of simpson diversity indices between different reproductive stages in sows. In all panels, boxes represent the interquartile range (IQR) between the first and third quartiles. The lines inside boxes represent the median. Whiskers denote the lowest and highest values within 1.5 IQR from the first and third quartiles, respectively. Sample size: n=12. a-b, significant effect of sampling time (*P* < 0.05; values with different lowercase letters are significantly different).


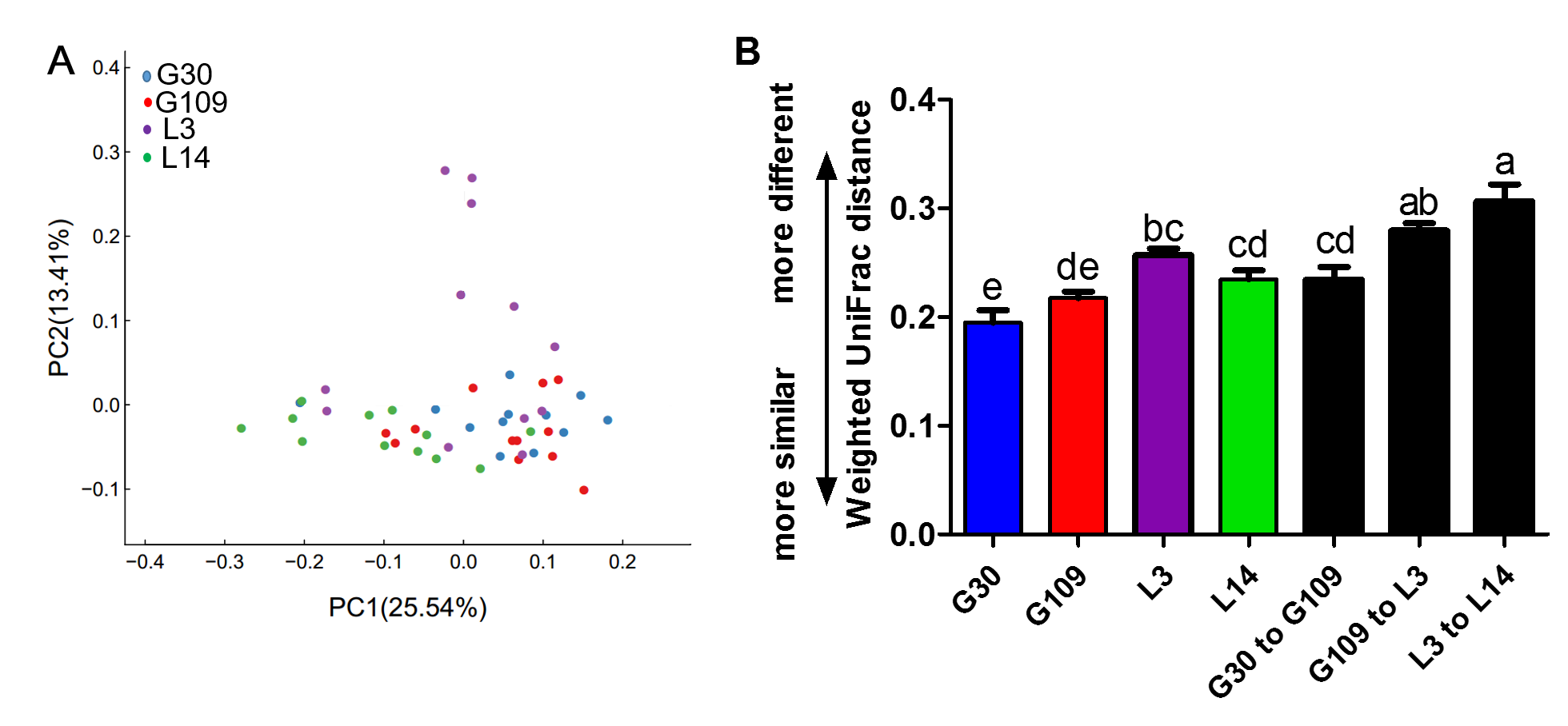


**Supplementary Figure 4. Inter- and intraindividual variations of the gut microbiota of the sows. (A)** PCoA based on weighted UniFrac distance. **(B)** Inter-and intraindividual variations of the gut microbiota based on weighted UniFarc distance among different reproductive stages in sows. Data are presented as means ± SEM (n =12). a-e, significant effect of sampling day (*P* < 0.05; values with different lowercase letters are significantly different). G109, day 109 of gestation; L3, day 3 of lactation.

## Supplementary Tables

Supplementary Table 1. Ingredients and nutrient composition of experimental gestation and lactation diets (as-fed basis)

| **Ingredients** | **Gestation diet** | **Lactation diet** |
| --- | --- | --- |
| Ingredient, % |  |  |
| Corn | 54.80 | 54.40 |
| Soybean meal, 43%CP | 11.50 | 26.00 |
| Wheat bran | 0.00 | 11.00 |
| Soybean hull | 14.00 | 0.00 |
| Rice bran meal | 16.00 | 0.00 |
| Calcium carbonate | 1.20 | 1.51 |
| Dicalcium phosphate | 1.00 | 1.23 |
| Salt | 0.40 | 0.26 |
| Mildewcide^1^ | 0.10 | 0.10 |
| Choline chloride | 0.00 | 1.00 |
| Premix^2^ | 1.00 | 1.50 |
| Nutrient composition |  |  |
| Net energy, MJ/kg^3^ | 9.53 | 10.36 |
| Crude protein, % | 13.96 | 18.92 |
| ISF, %^4^ | 26.95 | 14.45 |
| SF, %^5^ | 2.85 | 1.76 |
| NDF, %^6^ | 15.70 | 11.18 |
| ADF, %^7^ | 5.47 | 4.26 |
| Lys, %^3^ | 0.59 | 1.03 |
| Ca, %^3^ | 0.78 | 1.06 |
| Available P, %^3^ | 0.40 | 0.45 |

^1^Mildewcide: ammonium propionate.

^2^Provided per kg of diet: Cu, 30 mg; Fe, 160 mg; Zn, 160 mg; Mn, 55 mg; I, 0.5 mg; Se, 0.5 mg; Co, 0.8 mg; Cr, 0.2 mg; Vitamin A, 14000 IU; Vitamin D_3_, 2900 IU; Vitamin E, 120 mg; Vitamin K_3_, 6 mg; Vitamin B_1_, 2.4 mg; Vitamin B_2_, 8.5 mg; Vitamin B_6_, 4.5 mg; Vitamin B_12_, 0.03 mg; Vitamin H, 0.55 mg; Pantothenic acid, 30 mg; Folic acid, 5 mg; Nicotinamide, 50 mg.

^3^Calculated chemical concentrations using values for feed ingredients from the NRC (2012).

^4^insoluble fiber.

^5^solubel fiber.

^6^neutral detergent fiber.

^7^acid detergent fiber.

# Supplementary Table 2. The relative abundances of 26 distinct genera across the reproductive cycle were significantly different.^1^

| **Genera** | **The reproductive cycle^2^** | | | | ***P*-value** | **FDR** |
| --- | --- | --- | --- | --- | --- | --- |
|  | **G30** | **G109** | **L3** | **L14** |  |  |
| *Alloprevotella* | 0.51±0.17^b^ | 0.37±0.1^b^ | 0.26±0.07^b^ | 1.06±0.22^a^ | 0.0026 | 0.007 |
| *Anaerovibrio* | 0.11±0.06^b^ | 0.06±0.02^b^ | 0.09±0.04^b^ | 0.46±0.12^a^ | 0.0006 | 0.003 |
| *Bacillus* | 0.10±0.03^a^ | 0.08±0.05^ab^ | 0.00±0.00^b^ | 0.00±0.00^b^ | 0.0241 | 0.032 |
| *Butyricicoccus* | 0.04±0.01^b^ | 0.01±0.01^b^ | 0.05±0.01^b^ | 0.33±0.14^a^ | 0.0101 | 0.020 |
| *Campylobacter* | 0.19±0.06^a^ | 0.12±0.06^ab^ | 0.04±0.02^b^ | 0.02±0.01^b^ | 0.0206 | 0.028 |
| *Cellulosilyticum* | 0.30±0.10^ab^ | 0.10±0.03^b^ | 0.18±0.06^b^ | 0.63±0.24^a^ | 0.0359 | 0.043 |
| *Clostridium_sensu_stricto_1* | 1.99±0.58^b^ | 1.16±0.42^b^ | 7.16±3.30^a^ | 1.15±0.21^b^ | 0.0447 | 0.051 |
| *Lachnoclostridium* | 0.06±0.02^b^ | 0.03±0.01^b^ | 0.04±0.01^b^ | 0.18±0.04^a^ | 0.0002 | 0.001 |
| *Oribacterium* | 0.02±0.00^b^ | 0.02±0.01^b^ | 0.12±0.06^ab^ | 0.15±0.04^a^ | 0.0183 | 0.028 |
| *Phascolarctobacterium* | 1.81±0.43^a^ | 0.74±0.2^b^ | 0.66±0.26^b^ | 0.97±0.19^b^ | 0.0282 | 0.036 |
| *Porphyromonas* | 0.00±0.00^b^ | 0.00±0.00^b^ | 0.12±0.09^a^ | 0.00±0.00^b^ | 0.0002 | 0.001 |
| *Prevotella_1* | 1.16±0.25^b^ | 0.94±0.13^b^ | 1.25±0.63^b^ | 3.05±0.86^a^ | 0.0338 | 0.042 |
| *Prevotella_2* | 0.02±0.01^b^ | 0.03±0.01^b^ | 0.08±0.05^b^ | 0.25±0.08^a^ | 0.0023 | 0.007 |
| *Prevotellaceae_UCG-001* | 2.51±0.44^ab^ | 3.46±0.96^a^ | 1.02±0.39^b^ | 1.65±0.46^ab^ | 0.0391 | 0.046 |
| *Prevotellaceae_UCG-003* | 0.31±0.12^b^ | 0.36±0.19^b^ | 0.13±0.05^b^ | 0.77±0.13^a^ | 0.0125 | 0.024 |
| *Prevotellaceae_UCG-004* | 0.48±0.12^a^ | 0.41±0.1^a^ | 0.12±0.04^b^ | 0.24±0.06^ab^ | 0.0208 | 0.028 |
| *Quinella* | 0.58±0.11^a^ | 0.47±0.25^a^ | 0.03±0.01^b^ | 0.06±0.02^b^ | 0.0098 | 0.020 |
| *Ruminiclostridium_6* | 0.80±0.13^a^ | 0.34±0.1^b^ | 0.11±0.02^b^ | 0.19±0.02^b^ | 0.0000 | 0.000 |
| *Ruminococcaceae_NK4A214* | 2.12±0.11^a^ | 1.59±0.47^b^ | 2.3±0.33^b^ | 3.45±0.45^b^ | 0.0070 | 0.016 |
| *Ruminococcaceae_UCG-005* | 3.67±0.39^a^ | 2.55±0.53^ab^ | 1.75±0.34^b^ | 2.48±0.37^ab^ | 0.0205 | 0.028 |
| *Ruminococcaceae_UCG-009* | 0.07±0.01^b^ | 0.05±0.01^b^ | 0.05±0.01^b^ | 0.15±0.04^a^ | 0.0194 | 0.028 |
| *Ruminococcaceae_UCG-013* | 0.63±0.08^a^ | 0.41±0.04^b^ | 0.31±0.07^b^ | 0.62±0.07^a^ | 0.0023 | 0.007 |
| *Ruminococcaceae_UCG-014* | 3.83±0.67^a^ | 1.59±0.59^b^ | 0.58±0.19^b^ | 1.07±0.23^b^ | 0.0001 | 0.001 |
| *Ruminococcus_1* | 1.17±0.17^a^ | 0.57±0.22^b^ | 0.49±0.11^b^ | 0.46±0.06^b^ | 0.0044 | 0.011 |
| *Terrisporobacter* | 0.55±0.1^b^ | 0.43±0.17^b^ | 0.32±0.08^b^ | 1.13±0.18^a^ | 0.0007 | 0.003 |
| *Turicibacter* | 0.20±0.04^a^ | 0.10±0.03^bc^ | 0.06±0.02^c^ | 0.18±0.04^ab^ | 0.0137 | 0.024 |

^1^Data are presented as means ± SEM (n =12).

^2^G109, day 109 of gestation; L3, day 3 of lactation.

^a-c^significant effect of sampling day (values with different lowercase letters are significantly different).
